# Confirmation bias is adaptive when coupled with efficient metacognition

**DOI:** 10.1098/rstb.2020.0131

**Published:** 2021-04-12

**Authors:** Max Rollwage, Stephen M. Fleming

**Affiliations:** ^1^ Wellcome Centre for Human Neuroimaging, University College London, London WC1N 3BG, UK; ^2^ Max Planck University College London Centre for Computational Psychiatry and Ageing Research, London WC1B 5EH, UK; ^3^ Department of Experimental Psychology, University College London, London WC1H 0AP, UK

**Keywords:** confirmation bias, metacognition, belief updating, adaptive rationality, selective information processing

## Abstract

Biases in the consideration of evidence can reduce the chances of consensus between people with different viewpoints. While such altered information processing typically leads to detrimental performance in laboratory tasks, the ubiquitous nature of confirmation bias makes it unlikely that selective information processing is universally harmful. Here, we suggest that confirmation bias is adaptive to the extent that agents have good metacognition, allowing them to downweight contradictory information when correct but still able to seek new information when they realize they are wrong. Using simulation-based modelling, we explore how the adaptiveness of holding a confirmation bias depends on such metacognitive insight. We find that the behavioural consequences of selective information processing are systematically affected by agents' introspective abilities. Strikingly, we find that selective information processing can even improve decision-making when compared with unbiased evidence accumulation, as long as it is accompanied by good metacognition. These results further suggest that interventions which boost people's metacognition might be efficient in alleviating the negative effects of selective information processing on issues such as political polarization.

This article is part of the theme issue ‘The political brain: neurocognitive and computational mechanisms’.

## 1. Introduction

Polarization between opposing viewpoints is increasingly prevalent in discussions surrounding political and societal issues [1]. An important cognitive driver of this polarization is the human tendency to discount evidence against one's current position [2–5], a phenomenon known as confirmation bias [6]. Confirmation bias has been reported in a variety of settings [7], including the formation of clinical diagnosis [8], inference about people's character [9], investment decisions [10], views about societal issues such as capital punishment [11] and climate change [12]. Perhaps most prominently, confirmation bias has been reported in relation to politically charged beliefs, such that people are generally prone to process information in line with their political convictions [13–17]. On a societal level, such skewed information intake might lead to entrenched beliefs, and, in turn, the prevalence of dogmatic groupings and widespread polarization [5,18]. In line with this hypothesis, people who show a resistance to belief updating are also more likely to show extreme political beliefs [19], aggression towards opposing political views [20], and authoritarian [21] or dogmatic traits [4,22].

Recent cognitive neuroscience studies have identified the selective integration of choice-consistent information as a key mechanism underpinning this cognitive bias [23–25]. For instance, we recently showed that dogmatic participants were characterized by two cognitive alterations in the context of a perceptual decision-making task [4]. First, dogmatic participants showed a reduction in metacognitive ability, manifesting as a selective overconfidence after making errors. Second, metacognitive ability (i.e. the accuracy of confidence judgements) was predictive of post-decision evidence integration, where people with poorer metacognition showed less sensitivity for corrective information.

These results indicate that confidence may act as an internal control signal that guides future information processing [26–30]. In studies using magnetoencephalography, we found evidence for this hypothesis, with confidence strongly modulating the extent of neural post-decision processing. Evidence accumulation was largely unbiased after low confidence decisions but displayed a confirmation bias after high confidence decisions [24]. In other words, people appear especially resistant to corrective information when they are highly confident about a wrong decision. However, when confidence is well aligned with performance—when metacognitive ability is high—weighting post-decisional integration by confidence is likely to be less problematic, as people will tend to be less confident after making errors, and, therefore, also open to corrective information. This line of reasoning suggests that people's metacognitive ability might be a crucial driver of the degree to which selective information processing leads to negative behavioural outcomes.

Here, we test a hypothesis that selective information integration might be adaptive when coupled with high metacognitive ability. Our proposal is in line with a broader hypothesis that the ubiquitous nature of selective evidence integration makes it unlikely that this cognitive characteristic is always maladaptive [31,32]. For instance, others have interpreted confirmation bias as a heuristic that reduces computational complexity [33] or allows for robustness against noise [34,35]. Here, we offer an alternative perspective: that confirmation bias is adaptive to the extent it is accompanied by a metacognitive ability to effectively monitor and recognize when we might be wrong [36]. We use simulation-based modelling to compare different evidence integration strategies and test their respective performances. Specifically, we compared unbiased evidence integration with a simple confirmation bias as well as with a confidence-driven confirmation bias (as observed empirically in [24]). Because we expected the performance of a confidence-weighted confirmation bias to depend on the reliability with which confidence judgements indicate choice accuracy, we also investigated the influence of metacognitive ability on the adaptiveness of confirmation bias.

## 2. Modelling behavioural impact of selective evidence integration strategies

We model a simple situation in which agents make a binary decision between two choice options based on noisy information drawn from a world state that is unknown to the agent. This situation is easily accommodated by existing frameworks for characterizing belief updating [25,37,38], and closely resembles common perceptual decision-making paradigms. This setting also acts as a minimal framework within which more complex decision problems can be modelled. For instance, to reach an opinion about whether human activity causes global warming (the ground truth), we have to form beliefs based on multiple noisy evidence samples (e.g. scientific publications and newspaper articles). Importantly, this process requires the updating of pre-existing beliefs whenever more evidence becomes available. In such a situation, Bayesian belief updating is often used as benchmark model [39,40]. Thus, we incorporated a Bayesian updater as our ‘unbiased agent’ against which other evidence integration strategies can be compared. A Bayesian agent keeps track of its graded belief that one or other choice option is correct by calculating the probability of the chosen option, given the evidence, compared to the alternative: *P*(choice|evidence)/*P*(alternative|evidence)

For simplicity, we simulate a situation in which participants only receive two samples of information: *X*_pre_ represents the initial information and *X*_post_ represents the additional (or post-decision) evidence. Both *X*_pre_ and *X*_post_ are sampled from normal distributions:

$$X_{\mathrm{pre}}∼N(\mu ,\sigma_{\mathrm{pre}}^{2})$$

$$X_{\mathrm{post}}∼N(\mu ,\sigma_{\mathrm{post}}^{2})$$

withμ=[−1,1],

where the common mean (*µ*) of these distributions corresponds to the actual world state that needs to be inferred. While pre- and post-decision evidence distributions have the same mean (i.e. indicate the same underlying world state), they might differ in their variance. The variance represents the reliability of the information, with higher variances indicating less reliable information. We simulate different reliabilities of pre- and post-decision evidence as the consequences of a confirmation bias are likely to depend on this balance.

After receiving initial information, agents make an initial decision (decision_initial_) which depends solely on *X*_pre_. If *X*_pre_ has a positive value (*X*_pre_ > 0), decision_initial_ = 1, whereas if *X*_pre_ has a negative value (*X*_pre_ < 0), decision_initial_ = −1. An estimate of confidence in this initial decision is derived by calculating the log-odds in favour of the chosen world state:

$${\text{LO}}_{\mathrm{pre}}=\frac{2{\;}^{*}\;\mu {\;}^{*}\;X_{\mathrm{pre}}}{\sigma_{\mathrm{pre}}^{2}}.$$

These log-odds can be transformed into a probability of being correct (between 0 and 1) as follows:

$$\text{confidenc}{\text{e}}_{\mathrm{initial}}=\frac{{\text{e}}^{{\mathrm{LO}}_{\mathrm{pre}}}}{\left(1+{\text{e}}^{{\mathrm{LO}}_{\mathrm{pre}}}\right)}.$$

After the initial decision, the agent receives additional information in the form of *X*_post_. In order to reach a final decision, both evidence samples can be integrated in an unbiased Bayesian fashion by simply summing the log-odds:

$${\text{LO}}_{\mathrm{final}}={\text{LO}}_{\mathrm{pre}}+\;{\text{LO}}_{\mathrm{post}}.$$

Note that by summing the log-odds of pre- and post-decision evidence, the certainty/reliability of these two evidence samples is implicitly considered, i.e. evidence is combined in line with Bayesian principles. The final decision depends on the sign of the posterior log-odds (LO_final_). If the final decision corresponds to the actual state of the world, the agent can be said to have formed an accurate belief and performs the task correctly. We refer to this unbiased decision-maker as a Bayesian agent.

A confirmation bias can be modelled within this framework as an altered incorporation of post-decision evidence dependent on whether this new information confirms or disconfirms the initial decision:

if sign(*X*_post_) = sign (decision_initial_):

$${\text{LO}}_{\mathrm{final}}={\text{LO}}_{\mathrm{pre}}+(\text{1 + confirmation bias})\;*\;{\text{LO}}_{\mathrm{post}}.$$

else if sign(*X*_post_) ≠ sign (decision_initial_):

$${\text{LO}}_{\mathrm{final}}={\text{LO}}_{\mathrm{pre}}+(1-\text{confirmation bias})\;*\;{\text{LO}}_{\mathrm{post}}.$$

This form of confirmation bias can range from 0 (no bias) to 1 (where processing of disconfirmatory information is abolished).

In line with empirical observations, we also modelled a situation in which confirmation bias is modulated by confidence [24], such that participants were relatively unbiased in their use of new evidence when less confident, but showed an enhanced confirmation bias after high confidence decisions. To mimic these signatures, we simulated a ‘metacognitive’ agent which shows a confirmation bias when it is confident in an initial choice (confidence = 1), but remains unbiased when unsure (confidence = 0.5):

if sign(*X*_post_) = sign (decision_initial_):

$$\begin{array}{rl} {\text{LO}}_{\mathrm{final}} & ={\text{LO}}_{\mathrm{pre}}\\ & \;+(1+(\text{confirmation bias}\;*\;((\text{confidence}-0.5)\;*\;2))\\ & \;*\;{\text{LO}}_{\mathrm{post}}, \end{array}$$

else if sign(*X*_post_) ≠ sign (decision_initial_):

$$\begin{array}{rl} {\text{LO}}_{\mathrm{final}} & ={\text{LO}}_{\mathrm{pre}}\\ & \;+(1-(\text{confirmation bias}\;*\;((\text{confidence}-0.5)\;*\;2))\\ & \;*\;{\text{LO}}_{\mathrm{post}}. \end{array}$$

We note that a metacognitive agent differs from a Bayesian agent in that its initial confidence directly modulates the extent to which post-decision evidence is incorporated. For a Bayesian agent, there is of course also a sense in which confidence ‘weights’ the subsequent incorporation of evidence, in that a highly confident decision will require more disconfirming evidence to be overturned. Such updates, however, are in keeping with the linear accumulation of the log-odds of one or other hypothesis. By contrast, our metacognitive agent downweights the processing of disconfirmatory evidence when it is confident, representing a nonlinear effect of confidence on the incorporation of post-decisional log-odds. Thus, our metacognitive agent shows similarities with circular inference models [41,42], in which prior beliefs directly alter sensitivity to new evidence.

For each agent and each combination of evidence strength (see below), we simulated 200 000 trials, with the average decision accuracy across these simulated trials forming our measure of the agent's performance. In what follows, we describe in detail how these simulations were conducted.

## 3. Methods

### (a) Pre- and post-decision evidence strength

**Figure 1.**
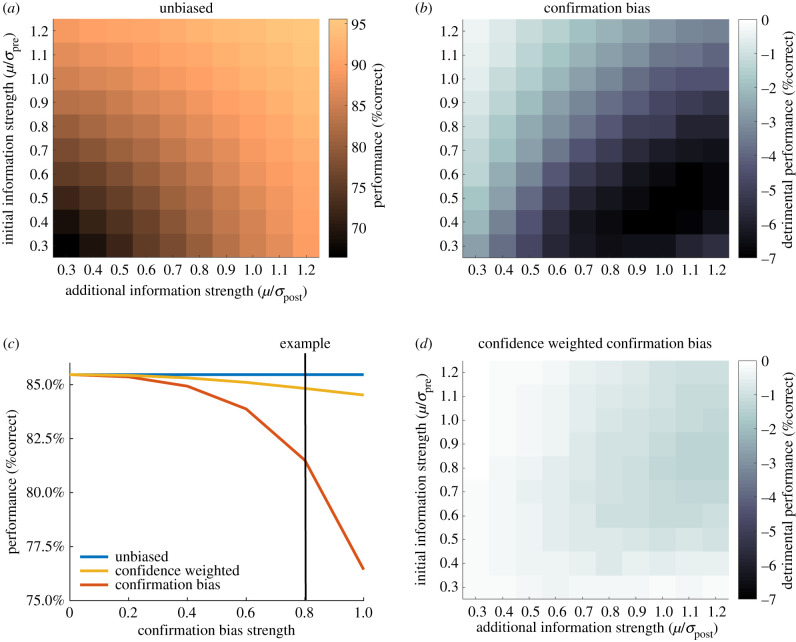
Comparison of agents' performance with different biases in information processing. (*a*) Performance of an unbiased agent that integrates initial and additional information in a Bayesian manner. Depending on the evidence strength, this agent shows different levels of accuracy, with better performance when both evidence samples are strong/reliable. (*b*) Difference in performance between an unbiased agent and a confirmation-biased agent as a function of the reliability of the initial and additional evidence. A confirmation bias has especially detrimental effects when initial evidence is relatively weak. (*c*) Comparison of unbiased, confirmation-biased and metacognitive (confidence-weighted) agents as a function of confirmation bias strength. Performance is averaged over all combinations of initial and additional evidence strengths. The vertical line indicates the strength of confirmation bias used in (*b*) and (*d*). (*d*) Difference in performance between an unbiased agent and a metacognitive agent as a function of the reliability of initial and additional evidence. Overall, the metacognitive agent shows only a relatively small disadvantage in comparison to an unbiased agent. In comparison to a simple confirmation bias, the metacognitive agent suffers less performance detriment in situations with weak initial evidence. (*b*,*d*) Dark colours indicate more detrimental performance of confirmation bias strategies when compared with unbiased evidence integration.

**Figure 2.**
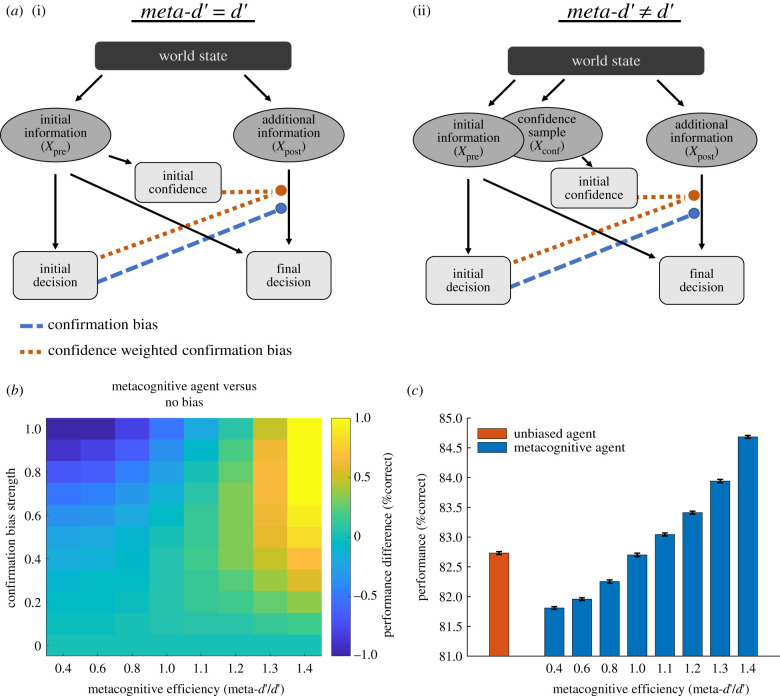
Performance of a metacognitive agent as a function of metacognitive efficiency. (*a*) Illustration of different decision and confidence models. (i) A model in which the same evidence (*X*_pre_) informs both the initial decision and the initial confidence, resulting in a ratio of meta-*d*′/*d*′ = 1. (ii) A situation in which meta-*d*′ and *d*′ can differ as the initial decision and confidence rely on separate, though correlated, evidence samples (*X*_pre_ and *X*_conf_). The final decision is determined by a combination of the initial (*X*_pre_) and the additional (*X*_post_) information. The coloured arrows indicate the way in which either an initial decision (confirmation bias) or an initial decision in combination with confidence (confidence-weighted confirmation bias, as in our metacognitive agent) modulate the incorporation of post-decision evidence. (*b*) Performance difference for a metacognitive agent compared to an unbiased agent. The performance of a confidence-weighted confirmation bias is sensitive to metacognitive efficiency (i.e. the accuracy of confidence ratings), with the greatest benefits obtained when metacognitive ability is high. When the ratio of meta-*d*′/*d*′ is above 1, a metacognitive agent outperforms an unbiased agent. Hotter colours indicate better performance of the metacognitive agent. (*c*) Performance of an unbiased agent compared to metacognitive agents differing in their metacognitive efficiency. Here, we fix the pre- and post-decision evidence strengths to an intermediate level (see Methods) in order to reveal differences between different decision strategies. We simulated 100 agents for each setting and present group means ± s.e.m. Metacognitive agents with lower metacognitive efficiencies (meta-*d*′/*d*′ < 1) show significantly lower performance than an unbiased agent (all *p* < 0.0001), whereas metacognitive agents with higher metacognitive efficiencies (meta-*d*′/*d*′ > 1) show significantly better performance than an unbiased agent (*p* < 0.0001). An agent with meta-*d*′/*d*′ = 1 yields performance that is not significantly different from that of an unbiased agent (*p* = 0.32).

As the performance of the different agents might depend on the reliabilities of pre- (*X*_pre_) and post-decisional (*X*_post_) information, we simulated different information strengths defined as *z*-scores (*μ*/*σ*). We fixed *µ* = 1 (or −1, respectively) and changed *σ*_pre_ and *σ*_post_ to vary information strength. We modelled all combinations of pre-decision (*μ*/*σ*_pre_ = [0.3–1.2]) and post-decision (*μ*/*σ*_post_ = [0.3–1.2]) information strengths. In figure 1*a*,*b* and *d*, analyses of the joint effects of these two evidence strengths are presented, whereas in figures 1*c* and 2*a*,*c*, performance is averaged over all evidence strength conditions.

### (b) Generative model of metacognitive abilities

The correspondence between confidence and performance can be formally quantified as the ratio of meta-*d′/d′* (known as metacognitive efficiency), where meta-*d′* reflects metacognitive sensitivity and *d′* reflects primary task performance within a signal detection theoretic framework [43]. Several reasons for a dissociation between meta*-d′* and *d′* have been suggested. For instance, confidence may reflect a noisy read-out of the decision evidence or a decline of decision evidence in working memory prior to a confidence judgement [43], leading to a meta*-d′*/*d′* ratio of less than 1. On the other hand, confidence might be informed by evidence that was not available at the time of decision [44–46], or on correlated evidence that is accumulated in parallel [47], both of which may lead meta*-d′/d′* ratios to surpass 1.

To model different degrees of metacognitive ability, we relaxed our assumption that confidence is directly derived from the evidence informing the initial decision (figure 2*a*). Instead, the evidence informing decisions (*X*_pre_) and confidence estimates (*X*_conf_) were modelled as distinct but correlated, and we allowed the reliability of the confidence ($\sigma_{\mathrm{conf}}^{2}$) and decision ($\sigma_{\mathrm{pre}}^{2}$) samples to differ. Specifically, *X*_pre_ and *X*_conf_ were sampled from a bivariate normal distribution with mean *µ* and covariance Σ:

$$\left[\begin{array}{c} X_{\mathrm{pre}}\\ X_{\mathrm{conf}} \end{array}\right]∼N(\mu ,\Sigma )$$

and

$$\Sigma =\left[\begin{array}{cc} \sigma_{\mathrm{pre}}^{2} & \rho \;*\;\sigma_{\mathrm{pre}}\;*\;\sigma_{\mathrm{conf}}\\ \rho \;*\;\sigma_{\mathrm{pre}}\;*\;\sigma_{\mathrm{conf}} & \sigma_{\mathrm{conf}}^{2} \end{array}\right].$$

Σ parametrizes the relationship between *X*_pre_ and *X*_conf_, with *ρ* representing the correlation between these variables. As described by Fleming & Daw [47], in this situation confidence can be inferred based on a combination of *X*_conf_, the initial decision and the covariance of *X*_conf_ and *X*_pre_ (see the appendix of [47], for further details on this calculation):

$$\text{confidence}=P(\mathrm{decisio}{\text{n}}_{\mathrm{initial}}=\mu |X_{\mathrm{conf}},\;\text{decisio}{\text{n}}_{\mathrm{initial}},\;\Sigma ).$$

Importantly, modelling separate samples of *X*_conf_ and *X*_pre_ allows for dissociations between meta*-d*′ and *d*′, thus making it possible to simulate varying degrees of metacognitive efficiency. For instance, a decrease in the reliability of the evidence informing the confidence rating ($\sigma_{\mathrm{conf}\;}^{2}>\;\sigma_{\mathrm{pre}}^{2}$) naturally reduces metacognitive efficiency and leads confidence judgements to be less reliable predictors of choice accuracy. To simulate lower metacognitive abilities (meta*-d′*/*d′* < 1), we fixed *ρ* = 0.8 and varied $\sigma_{\mathrm{conf}\;}^{2}$(however, we note that our findings are not dependent on the specific value of *ρ* or $\sigma_{\mathrm{conf}\;}^{2}$; see the electronic supplementary material, figure S1). $\sigma_{\mathrm{conf}\;}^{2}$was defined by multiplying $\sigma_{\mathrm{pre}}^{2}$ by the set of coefficients [2.04, 1.56, 1.23], which were selected to result in ratios of meta*-d′*/*d′* of [0.4 0.6 0.8]. We also modelled a situation in which confidence and decision information have the same reliability ($\sigma_{\mathrm{pre}}^{2}=\sigma_{\mathrm{conf}}^{2}$), but the evidence samples have a variable degree of correlation (*ρ* = [0.1, 0.3, 0.5, 0.65]), resulting in values of meta*-d′*/*d′* of [1.4, 1.3, 1.2, 1.1]. Such decorrelations in evidence samples result in increased metacognitive efficiency because there is additional information on which to base an evaluation of the decider [47].

### (c) Assessment of metacognitive efficiency

To assess whether manipulations in our generative model of confidence had the intended influence on agents' metacognitive efficiency, we calculated the meta*-d′*/*d′* ratio for each agent [43] based on their simulated behaviour, using the MLE toolbox of Maniscalco and Lau (http://www.columbia.edu/~bsm2105/type2sdt/). Model fits were conducted separately for each level of evidence strength and then averaged over all evidence strengths for each agent.

### (d) Robustness and significance

We simulated many trials per condition (200 000 trials for each combination of pre- and post-decision evidence strength) to ensure robustness to noise perturbations. We also sought to provide a statistical test of the modulation of performance by metacognitive efficiency. We simulated 100 agents per metacognitive efficiency setting (with confirmation bias = 1) and compared their performance to an unbiased agent (also simulated 100 times) using a *t*-test. We also evaluated the effects of metacognitive efficiency on post-decision performance at intermediate pre-decision evidence *μ*/*σ*_pre_ = [0.8] and post-decision evidence *μ*/*σ*_pre_ = [0.5] strengths, which are similar to those commonly used in previous laboratory experiments.

## 4. Results

Depending on the different reliabilities of *X*_pre_ and *X*_post_, an unbiased Bayesian observer achieves different final performances (figure 1*a*), with more reliable information yielding better performance. In addition to an unbiased observer, we also modelled agents with gradually increasing levels of confirmation bias (figure 1*c*). As hypothesized, selectively accumulating confirmatory information results in detrimental performance, with higher levels of confirmation bias leading to a more pronounced detriment. At higher levels of confirmation bias, a detriment in performance from 85% correct to approximately 77% correct was observed, which is significant in a 2 alternative forced choice scenario where performance can only vary between 50 and 100% and evidence strength is adjusted to be of intermediate difficulty. As expected, this effect was most notable when the agent received relatively weak initial information but reliable post-decision evidence (figure 1*b*), as the bias prevents the incorporation of more reliable corrective information.

We next examined the performance of a metacognitive agent with a confidence-weighted confirmation bias. Notably, a metacognitive agent outperforms a simple confirmation bias in all settings and only shows slight impairments in relation to an unbiased agent (figure 1*c*). We also found that a metacognitive agent clearly outperforms a simple confirmation bias in situations when the initial evidence is weak (figure 1*d*). While a simple confirmation bias has the strongest decrement in performance in these situations, a metacognitive agent ‘realizes’ it is dealing with weak initial evidence (by assigning lower confidence to these decisions) and thus shows a more equal sensitivity to confirming and disconfirming information.

Up until now we have assumed that agents calculate confidence in an initial choice by directly evaluating the reliability of evidence that informed the decision, resulting in a fixed metacognitive efficiency (meta*-d′*/*d′* = 1). However, empirical evidence shows that people differ in their metacognitive ability [4,48,49]. Thus, we next assessed the degree to which metacognitive efficiency influences the performance of our metacognitive agent.

We found that reduced metacognitive efficiency (meta*-d′/d′* < 1) leads metacognitive agents to show impaired performance compared to unbiased agents (all *p*-values < 0.0001, see cooler colours in figure 2*b*), as the weighting of new information by confidence becomes less effective. Conversely, when metacognitive efficiency is high (meta-*d′/d′* > 1), we found that a metacognitive agent can even outperform an unbiased agent (all *p*-values < 0.0001, see hotter colours in figure 2*b*). This result is striking as it suggests that a selective integration of information is not necessarily a ‘bias’ but represents an advantageous strategy for achieving optimal performance in the context of a realistic cognitive architecture (i.e. one in which metacognition is particularly efficient). Figure 2*c* displays the effect of metacognitive efficiency on the performance of a metacognitive agent at a fixed level of intermediate evidence strength, relative to an unbiased agent. While the magnitude of these differences might appear relatively small (of the order of 2–3% correct), individual differences between human participants performing a similar task cover a similar range (e.g. in [24] the standard deviation of performance across participants was 3.7% correct decisions).

## 5. Discussion

Here, we investigated the effects of confirmation bias on the accuracy of belief formation. Our central proposal is that when confirmation bias is a feature of a self-aware (metacognitive) agent, it ceases to be detrimental, and may even become adaptive. We used simulation-based modelling to compare the performance of agents with different forms of confirmation bias against an unbiased agent. A simple (non-metacognitive) confirmation bias showed detrimental effects compared to an unbiased agent in all settings. In comparison, a metacognitive agent which modulates the degree of confirmation bias by confidence (as documented empirically in human observers; [24]) outperformed a simple confirmation bias agent, and was in many cases not substantially worse than an unbiased agent. The benefit of weighting a confirmation bias by confidence is that when confidence is low, and errors are more likely, the system becomes open to new and potentially corrective information.

In turn, by simulating varying degrees of metacognitive efficiency, we found that the performance of our metacognitive agent was sensitive to its level of self-awareness. Strikingly, a metacognitive agent with high self-awareness could in some cases even outperform an unbiased agent, indicating that selective information processing might be particularly adaptive when coupled with good metacognitive abilities. These results are in accordance with a view that cognitive biases may have originally evolved for good evolutionary reasons and are often adaptive when considered in the context of the agent's environment, including its broader mental toolkit [31].

Why should high degrees of metacognitive efficiency be advantageous in this case? The core mechanism appears to be the capacity of a confidence estimate to provide a ‘second look’ on a decision, similarly to how an external adviser might give us a separate view on a topic. The benefit of this mechanism depends on the agent's metacognitive ability—as agents with good metacognition provide the most effective ‘internal’ advisory signals. Interestingly, however, a confidence-weighted confirmation bias outperformed a simple confirmation bias in all settings, even when metacognitive ability was relatively low (see the electronic supplementary material, figure S2), suggesting that the mere presence of confidence weighting, rather than acute metacognition *per se*, may be sufficient to avoid the most deleterious effects of confirmation bias.

While these advantages for metacognitive agents were relatively small in size, they were robust and similar in magnitude to individual differences in performance on comparable laboratory tasks (e.g. [24]). We note that here we modelled a situation in which only two consecutive samples of evidence had to be integrated. Even in this minimal paradigm, increases in metacognitive efficiency could lead to a 2–3% increase in the number of correct decisions. Such a bias towards higher performance on individual, isolated judgements is likely to be magnified in situations requiring the integration of multiple information samples over time. In such situations, even small alterations in information processing might summate over time and lead to substantial changes in belief accuracy.

By incorporating confirmation bias as part of a broader cognitive architecture in which different mental processes can interact with each other (e.g. decisional and metacognitive processes), selective information processing may become adaptive when compared with the same ‘bias’ considered in isolation. In the same spirit, it has been argued that heuristics that may appear as biases in simple and constrained laboratory tasks become beneficial in more complex environments [50]. More broadly, our study indicates that considering cognitive biases in isolation from other mental processes might lead to the wrong conclusions about the impact of a particular cognitive feature on behaviour.

We note that a behavioural benefit for a confidence-weighted confirmation bias over an unbiased agent was only present when simulating agents with ‘hyper’ metacognitive efficiency (i.e. meta*-d′/d* > 1), such that metacognition became more acute than first-order task performance. This might seem odd at first glance, as it implies that the system is not using all the information available to it at the time of making an initial choice, and only afterwards becomes more sensitive to whether it was right or wrong. However, this kind of pattern is commonly observed in empirical data [45,47], and is thought to be driven either by additional post-decisional processing, differences in the variance of signal compared to noise [51] or (as simulated here) parallel streams of information processing that allows the system to detect and correct its errors [45]. The capacity for rapid error detection is well established in human studies [30,52–55] and thus it is reasonable to assume that hyper metacognitive efficiency may be common in the healthy population.

Importantly, a metacognitive system does not need to be more reliable than the decision-maker to achieve high metacognitive ability: it needs only to incorporate partially independent information (as used here; see the electronic supplementary material, figure S1 for simulations using a wider set of parameters). In this respect, our results also contribute to a debate over why it might be useful for the brain to encode a confidence signal separately from representations of decision evidence [56,57]. A metacognitive agent that can realize its own mistakes (and assign low confidence to these decisions) will tend to become more open to new information owing to the confidence weighting applied to selective information processing (interestingly our model predicts that when confidence falls below 0.5 in a two-choice scenario, metacognitive agents should even show a ‘disconfirmation’ bias, and be more prone to seek out information contradicting their current position). Our results suggest that this benefit is only accrued when metacognition is partly independent of first-order cognition.

Selective information processing has been assumed to lead to skewed, entrenched and potentially inaccurate beliefs about a range of societal and political issues [5,18]. However, the current results suggest that the detrimental effects of selective information processing depend on people's broader self-awareness. In turn, metacognitive deficits might represent core drivers of polarized or radical beliefs, owing to their consequence for maladaptive confirmation bias. Interestingly, this hypothesis is in line with empirical observations [4], showing that more dogmatic participants show reduced metacognitive sensitivity which in turn is predictive of reduced post-decision evidence processing.

Recognizing metacognition as a central driver of belief polarization may make it possible to develop new strategies for debiasing decision-making [5]. The contributors to confirmation bias in any given setting are likely to be multifactorial, with more proximal causes (such as measures of cognitive style) having large effect sizes, but providing more limited mechanistic insight. By contrast, identifying small, reliable effect sizes associated with underlying mechanisms (such as the impact of confidence weighting) may bring us closer to the potential for targeted intervention. Excitingly, there are existing interventions that have been shown to boost people's metacognitive ability [58,59]. Our results indicate that cognitive training which improves domain-general self-awareness and metacognitive efficiency may help to alleviate the negative behavioural outcomes of selective information processing, and foster resilience against misinformation and belief polarization.

## Data accessibility

Code of all simulations for this study is available at a dedicated Github repository (https://github.com/MaxRollwage/ConfirmationBias_simulation).
